# A Case of Resection of Shoulder-Joint

**Published:** 1864-06

**Authors:** William Alexander Greene

**Affiliations:** Chief Surgeon Artillery, Third Army Corps, Army of Northern Virginia of the Confederate States.


Art. V.—*A Case of Resection of the Shoulder-Joint.* By William Alexander Greene, Chief Surgeon Artillery, Third Army Corps, Army of Northern Virginia of the Confederate States.

In none of the joints is resection more frequently called for, and in none has it been attended with more beautiful results, than that of the shoulder. There have been a variety of processes devised for this operation. Those of M. Malgaigne and M. Bourgery for the longitudinal incisions, and those of Syme and Bent for the flap operations, seem to possess the highest advantages, and result in the most good to the patient. I think those with the longitudinal incisions merely, are to be preferred whenever circumstances will admit.

The fact of this operation having been so often deferred by our field surgeons, because of apparent lack of facilities and meagre appliances, together with the many cautions contained in some of our recent works on military surgery with reference to operating "upon the field proper," has induced me to report a case operated upon under as unfavorable circumstances as we will often meet with, and with most satisfactory results.

Sergeant W. Hammond, aged 30 years, of the Purcell Battery, Pegram's battalion artillery, Third Army Corps, Army Northern Virginia, was wounded at the battle of Gettysburg, Pennsylvania, 2d of July, 1863, by a Minie ball entering near the posterior fold of the arm-pit of the left arm, passing through, lacerating the capsule, and producing extensive fracture of the humerus, splitting the bone from the surgical neck downwards for over three inches. He was brought from the battle ground immediately to the field hospital. There being no symptoms of vital depression and the patient in unusual good spirits, an operation was at once determined upon.

*Operation.*—Chloroform was administered until complete anæsthesia was induced. The patient being placed in the semi-recumbent position, I began by making an incision from the top of the coraco-clavicular triangle, and extending downwards for about five inches, dividing at one stroke the skin, the deltoid, and the capsule. The joint was thus freely exposed on the inner and upper surfaces, which gave great facility in the extraction of the head. *The long head of the biceps was divided*, and the insertion of the four articular muscles carefully cut with a probe-pointed bistoury. But finding great difficulty in extracting the head of the bone, which was much comminuted, I added to this longitudinal incision of Malgaigne a transverse cut, according to the modification of Textor, which gave to the external wound the shape of the letter L. The soft parts were then carefully separated from the bone, and the spiculæ imbedded in the surrounding muscles removed, and the shaft divided with the ordinary saw as far down as injured. The operation was completed by ligature of a single artery, and the incision closed by the interrupted suture. The arm was placed in an improved apparatus, similar to that used for fractured clavicle. He was placed upon the floor of the barn in which I had my hospital, with a *few straws* under him, and no "pillows to support his arm." He remained there until the evening of the 4th of July.

When General Lee's army began to fall back from Gettysburg, at his own request, he arose from his bed of straw unassisted, getting into a rough army wagon. After proceeding several miles, the ambulance train was halted for a few hours, when, in attempting to get from the wagon, he fainted and fell to the earth. He was lifted up and borne by some comrades to a barn near by, and stimulants and coarse nourishments administered. I advised him to remain where he was, and trust to Nature and the generosity of the Yankees for recovery.

He was then left, and heard from no more until exchanged; arriving in Richmond September 28th, 1863, less than three months from date of the operation, and having traveled from Gettysburg, Pennsylvania. The disadvantages under which he labored, and the suffering to which he was subjected, in common with many other noble fellows who spilled their blood freely at Gettysburg, as well as his present condition, &c., may be gathered from the following extracts of a letter received from him a few days since:

"I received no medical treatment from the time I was left (July 4th, near Salem,) more than an inexperienced nurse could give, until the 21st of July, when I was moved to Gettysburg, (which was nineteen days after the operation had been performed.) During all the time I used cold water applications alone. By the 1st of August I was able to sit up without assistance, and by the 15th could walk with ease. I steadily improved, and on the 10th of September was moved to Baltimore. Transportation very rough. On the 25th of September I was put aboard a steamer, in company with three hundred other badly wounded, all crowded on deck, exposed to cold night wind, thinly clad, and barely room to sit down. Thus I remained for three days, when I arrived in Richmond. Remained there from the 28th of September to the 8th of October. After my arrival home I was attacked with chills and fever, and as soon as they were stopped, my improvement was very rapid. Just six months from the time of the reception of my wound and the operation, I was entirely well. Nearly four inches of bone was taken from my arm, including the *head*, and yet I have *perfect* use of my hand and forearm. These functions not the least impaired. My arm is somewhat smaller, and shoulder slightly shrunken. I am now in enjoyment of almost perfect health, with every prospect of a long life." In another portion of his letter he says: "On the 15th of August I was visited by Dr. Jaynes, of Philadelphia, Surgeon-General of Pennsylvania, who examined my arm closely, and evinced much surprise that a rebel surgeon could perform a difficult operation so successfully." How notoriously consistent with Yankee character is the attempt of a Northern brother to defame the reputation and underrate the skill of Confederate surgeons, and that, too, by one *Jaynes*, a name most prominent of all others as having practised the most palpable absurdities and deceptions upon his fellow creatures by dispensing *secret nostrums—mystery* alone giving them value or importance, and implying the most disgraceful ignorance and fraudulent avarice.

